# Letter from the Editor in Chief

**DOI:** 10.19102/icrm.2022.130707

**Published:** 2022-07-15

**Authors:** Moussa Mansour


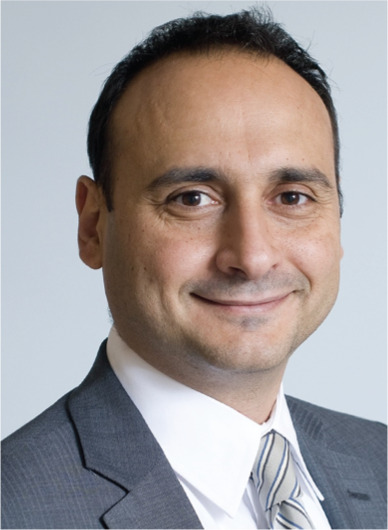


Dear readers,

Pulsed-field ablation (PFA) for cardiac arrhythmias was recently developed to incorporate a novel, non-thermal energy source for ablation. The procedure consists of applying rapid electrical pulses to the target area, resulting in apoptosis, without the use of thermal energy. Moreover, it is cardiac-specific and was demonstrated to spare collateral structures such as the esophagus and phrenic nerve. Single-arm studies were conducted in Europe and demonstrated its safety and efficacy, leading to the commercial release of PFA catheters there. In the United States, the technology remains investigational, awaiting the completion of randomized clinical trials that are currently ongoing.

Despite the large number of clinical and pre-clinical studies investigating PFA, some aspects of its mechanism of action remain poorly understood. One particular area is its effect on cardiac ganglionated plexi. This issue of *The Journal of Innovations in Cardiac Rhythm Management* contains an interesting article investigating this aspect of PFA, titled “Open-chest Pulsed Electric Field Ablation of Cardiac Ganglionated Plexi in Acute Canine Models.”^1^ In it, the authors demonstrated that the epicardial ablation of ganglionated plexi using pulsed-field energy results in altered markers of cardiac autonomic tone.

The ablation of ganglionated plexi has the potential to improve the success rate of catheter ablation if used as an adjunctive treatment to pulmonary vein isolation in selected patients; thus, it is possible that this study by van Zyl et al. may have important clinical implications in the future. However, it also has limitations, the most important of which is the fact that it was acute and thus lacks data concerning the chronic effect of PFA on the autonomic nervous system. It is well known that PFA can cause reversible stunning of the phrenic nerve when it is inadvertently ablated during pulmonary vein isolation. As a result, it is important to prove in longer-term studies that ablation of the ganglionated plexi is irreversible before it can be tested in human studies.

I hope that you find the content of this issue educational.


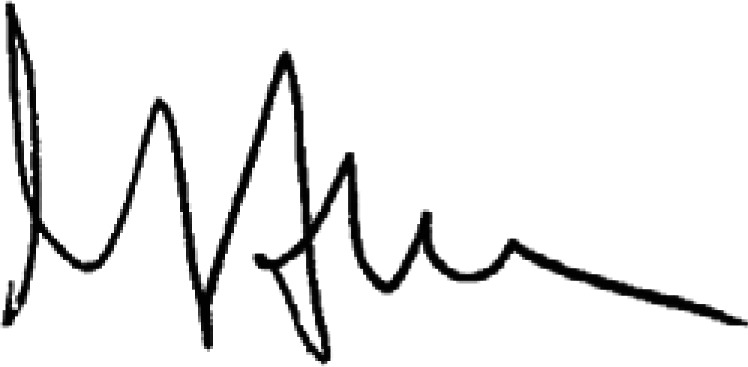


Sincerely,

Moussa Mansour, md, fhrs, facc

Editor in Chief

*The Journal of Innovations in Cardiac Rhythm Management*

MMansour@InnovationsInCRM.com

Director, Atrial Fibrillation Program

Jeremy Ruskin and Dan Starks Endowed Chair in Cardiology

Massachusetts General Hospital

Boston, MA 02114
